# Nucleoside analogs assisted with Chinese compound prescription in treating hepatic fibrosis of chronic hepatitis B patients: A protocol of systematic review and meta-analysis

**DOI:** 10.1097/MD.0000000000021032

**Published:** 2020-07-02

**Authors:** Maoyuan Cheng, Xianrong Feng, Long Wang, Yu Yang, Li Ma, Baojia Wang

**Affiliations:** a School of Basic Medicine, Chengdu University of Traditional Chinese Medicine; b Hospital of Chengdu University of Traditional Chinese Medicine, Chengdu, Sichuan, China.

**Keywords:** Chinese compound prescription, entecavir, tenofovir disoproxil fumarate, hepatic fibrosis, chronic hepatitis B

## Abstract

**Background:**

Chronic hepatitis B is often complicated with different degrees of hepatic fibrosis, which affects the quality of life. Nucleoside analogs are recommended by almost all guidelines in the world for the treatment of chronic hepatitis B. At present, there is no specific and effective chemical and biological agents for hepatic fibrosis. In China, Chinese compound prescription combined with nucleoside analogs have been used to treat hepatic fibrosis of chronic hepatitis B patients in more and more cases, and good results have been achieved. Several Chinese compound prescriptions that have been made into proprietary Chinese medicine for the convenience of use. This article aims to systematically evaluate the efficacy and safety of Chinese medicine compounds assisting nucleoside analogs in the treatment of hepatic fibrosis in chronic hepatitis B patients.

**Method:**

The following databases will be searched from their inception to September 2019: PubMed, EMBASE, EBSCOhost, The Cochrane Library, China National Knowledge Infrastructure (CNKI), Chinese Biomedical literature Database (CBM), VIP Database, Wanfang Database. Languages are limited to Chinese and English. The study includes randomized controlled trials using Chinese compound prescription combined with entecavir and Chinese compound prescription combined with tenofovir disoproxil fumarate to treat hepatic fibrosis of chronic hepatitis B patients. The primary outcomes including effective rate and biochemical parameters (levels of hyaluronic acid, laminin, pre-type-III collagen and type IV collagen will be tested. Additional outcomes include liver function indexes (levels of alanine aminotransferase, aspartate aminotransferase, total bilirubin) and levels of hepatitis B virus DNA. Stata14.0 software will be used for meta-analysis.

**Result:**

The efficacy and safety of Chinese compound prescriptions assisting nucleoside analogs for hepatic fibrosis of chronic hepatitis B patients will be assessed from the effective rate, biochemical parameters, liver function indexes, and levels of hepatitis B virus DNA.

**Conclusion:**

The conclusion of this study will be used to evaluate the efficacy and safety of Chinese compound prescriptions assisting nucleoside analogs in the treatment of hepatic fibrosis of chronic hepatitis B patients, as well as the adjuvant effectiveness of Chinese compound prescriptions in combined therapy.

**PROSPERO registration number:**

CRD42020156859.

## 1 Introduction

Hepatic fibrosis is a pathological change in various chronic liver diseases,^[1]^ which affects the normal physiological function of the liver. Chronic hepatitis B is a global public health problem. According to a World Health Organization survey, there are about 300 million people infected with chronic hepatitis B virus (HBV).^[2]^ Chronic hepatitis B patients often suffer from different degrees of hepatic fibrosis, which affects their quality of life and survival time. Long term anti HBV treatments, such as nucleoside analogs, can effectively inhibit the replication of HBV, reduce the inflammatory response of liver and achieve the effect of anti hepatic fibrosis.^[3]^ Although modern medicine has made some significant progress, there is no specific and effective chemical and biological agents for hepatic fibrosis. In clinical practice, Chinese compound prescriptions show its superiority in treating hepatic fibrosis.^[4]^ Traditional Chinese medicine (TCM), one of the important means to treat hepatic fibrosis, can inhibit the development of liver fibrosis through multiple mechanisms at multiple-lever.^[5]^ TCM practitioners’ understanding of hepatic fibrosis in chronic hepatitis B patients is based on the theories of TCM and their rich experience in treating hepatic fibrosis of chronic hepatitis B patients. In China, Chinese compound prescriptions combined with nucleoside analogs have been used to treat hepatic fibrosis of chronic hepatitis B patients in more and more cases, and good results have been achieved.^[6,7]^ Several kinds of compound prescriptions have been made into proprietary Chinese medicines according to national standards for the convenience of doctors and patients. However, there is a lack of systematic review and meta-analysis of Chinese compound prescriptions assisting nucleoside analogs in treating hepatic fibrosis of chronic hepatitis B patients. This protocol aims to systematically evaluate its effectiveness and safety, as well as its adjuvant role in the combined treatment.

## 2 Method

This protocol has been registered in PROSPER. It will be carried out following the Cochrane Handbook for Systematic Reviews of Interventions version 6.0^[8]^ and reporting will be done according to the guidelines of Preferred Reporting Items for Systematic Reviews and Meta Analyses.^[9]^

### 2.1 Inclusion criteria

#### 2.1.1 Types of study

Articles about Chinese compound prescriptions combined with nucleoside analogs (entecavir [ETV] or tenofovir disoproxil fumarate [TDF]) in the treatment of patients with hepatic fibrosis of chronic hepatitis B are selected according to the Cochrane Collaboration's randomized controlled trial criteria. Regardless of whether the blind method is used or not. Languages are limited to Chinese and English.

#### 2.1.2 Study participants

Patients meeting the diagnostic criteria of chronic hepatitis B, having antiviral indications and symptoms of liver fibrosis are included. The diagnosis of chronic hepatitis B should meet the criteria of the country where the patient is located, because different countries and regions define hepatitis B in slightly different ways.^[10–14]^ The diagnosis of hepatic fibrosis should meet the Consensus on the Diagnosis and Therapy of Hepatic Fibrosis (2019).^[15]^ Patients younger than 15 and older than 85 or with other serious diseases such as serious cardiovascular, cerebrovascular, endocrine, and hematological diseases will be excluded.

#### 2.1.3 Intervention

Intervention for the experimental group is a kind of nucleoside analogs combined with a Chinese compound prescription. The nucleoside analogs, recommended by almost all the guidelines in the world, are ETV and TDF.^[10–14]^ A Chinese compound prescription is composed of 2 or more TCMs in the form of decoction, tablet, pill, powder and paste, but not in the form of extract. Exclude any form of Chinese medicine extract. No other anti-liver fibrosis and antiviral treatment measures are applied. The control group are treated with ETV or TDF (a placebo can be used simultaneously). The treatment time was 12 weeks or more.

#### 2.1.4 Outcomes

##### 2.1.4.1 Primary outcomes

(1) Efficacy rate: clinical outcomes are divided into ineffective, effective, and significantly effective. The proportion of effective and significantly effective among the total number is efficacy rate.
(2) Biochemical parameters: levels of hyaluronic acid, laminin, pre-type-III collagen and type IV collagen (IV-C).

##### 2.1.4.2 Additional outcomes

(1) Liver function indexes: levels of alanine aminotransferase, aspartate aminotransferase, and total bilirubin.
(2) Levels of HBV DNA.

### 2.2 Search strategy

The following databases will be searched from their inception to September 2019: PubMed, EMBASE, EBSCOhost, The Cochrane Library, China National Knowledge Infrastructure (CNKI), Chinese Biomedical literature Database (CBM), VIP Database, Wanfang Database. There are no restrictions on the language of publication. We also manually searched relevant references mentioned in systematic reviews published previously. The following key words or phrases and their abbreviations or synonym are utilized singly or in combination: “Chinese compound prescription” or “Chinese compound formula” or “Chinese herbal medicine” or “Chinese medicine” or “traditional Chinese medicine” or “TCM” or “Chinese drugs” or “Chinese material medica” or “entecavir” or “ETV” or “tenofovir disoproxil fumarate” or “TDF” or “hepatic fibrosis” or “liver fibrosis” or “HBV” or “hepatitis B virusor” or “chronic hepatitis B” or “CHB.”

### 2.3 Studies Selection

**Figure 1:**
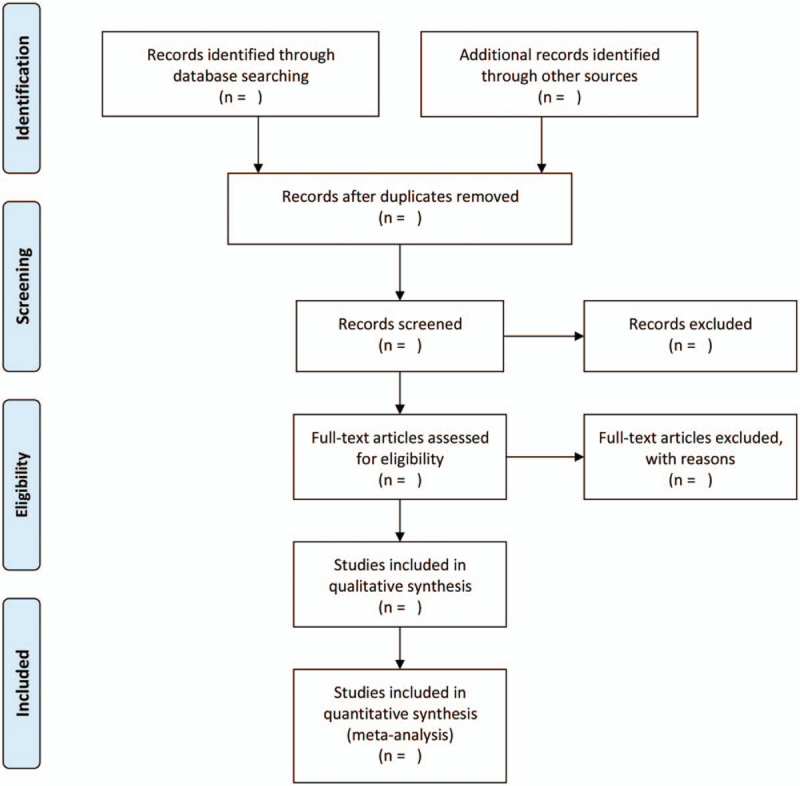
Preferred Reporting Items for Systematic Reviews and Meta-Analyses flow chart of study selection process.

Two reviewers (Maoyuan Cheng and Xianrong Feng) independently browsed the title and the abstract of every article to decide its relevance. The full text of the qualified articles will be investigated and then will be selected. The reasons for excluding any article should be noted down. Disagreements will be resolved through discussion between the 2 reviewers. If necessary, exact information will be sought from the author of the article. If they cannot reach a consensus, the third reviewer (Yu Yang) will make a final decision. The specific procedure of article selection is shown in Figure 1.

### 2.4 Data extraction

After determining which articles are finally included, an excel data extraction form will be designed which includes basic information (such as title, author, publication date, literature), general data (such as number of patients, source, gender, age), intervention (such as the name of medications, intervention period, intervention dosage), intervention effect (including various outcome indicators). The excel data extraction form will be collected independently by the 2 reviewers (Maoyuan Cheng and Xianrong Feng). They will cross-check each other's work. Differences will be resolved through discussion. If they do not reach a consensus, the third reviewer (Yu Yang) will make a final decision.

### 2.5 Risk of bias (quality) assessment

Two reviewers (Maoyuan Cheng and Xianrong Feng) will assess the risk of according to the Cochrane Handbook for Systematic Reviews of Interventions independently.^[8]^ The following items are included: random sequence generation, allocation concealment, blinding of participants and physicians, blinding of outcome assessment, incomplete outcome data, selective reporting and other sources of bias. Each of them will be classified as “high risk of bias,” “some concerns” or “low risk of bias.” Any disagreements will be resolved by the third reviewer (Yu Yang).

### 2.6 Strategy for data synthesis

The statistical data will be analyzed with the help of stata14.0. Risk ratios will be represented for dichotomous outcomes and weighted mean difference for continuous data. Ninety-five percent of the confidence interval will be calculated as an effective size. Homogeneity will be measured by the *I*^2^ and Cochran Q. Data will be analyzed with a random-effect model if the *I*^2^ > 50% or *P* < .10, which means high heterogeneity.

Otherwise, the fixed-effect model will be chosen if the *I*^2^ < 50% or *P* > .10, which means low heterogeneity.

### 2.7 Analysis of subgroups or subsets

Two prespecified subgroup analyses will be considered.

Subgroup 1: Chinese compound prescription and ETV vs ETV.

Subgroup 2: Chinese compound prescription and TDF vs TDF.

### 2.8 Sensitivity analysis

Sensitive analysis will be carried out when the heterogeneity between studies remains high after using subgroup analysis. Sensitivity analysis will be performed to identify the robustness of outcomes according to the following criteria: sample size, quality of the methods, and quality of the studies.

### 2.9 Assessment of reporting biases

More than 10 articles are contained in a meta-analysis, and funnel plot will be used to evaluate the risk of publication bias.

### 2.10 Quality of evidence

The quality of evidence will be assessed according to the Grading of Recommendations Assessment, Development and Evaluation system.^[16]^ Based on this grading systems, the result will be evaluated as “very low,” “low,” “moderate” or “high.”

### 2.11 Ethics

This research is a systematic review that does not contain personal information of patients. Therefore, informed consent and ethical permission are not required.

## 3 Discussion

For chronic hepatitis B patients with hepatic fibrosis, proper and prompt treatment, may prevent their conditions from developing into cirrhosis and even liver cancer, thus improves their quality of life and prolong their survival time. Nucleoside analogs are generally recognized as anti-HBV drugs. ETV and TDF are recommended by World Health Organization, as they can effectively prevent replication of HBV.^[13]^ The treatment of hepatic fibrosis should target the cause of the disease. However, with many differences and controversies in clinical and development research, there are no recognized specific and effective chemical and biological agents at present. In China, Chinese compound prescriptions are widely used in clinical practice for hepatic fibrosis. TCM believes that the pathogenesis and pathomechanism of hepatic fibrosis is the deficiency of vital energy and the invasion of evil Qi, which leads to qi stagnation and blood stasis. Clinical, experimental and evidence-based medicine studies show that the combination of Chinese compound prescription and nucleoside analogs is better than nucleoside analogs alone in treating hepatic fibrosis in chronic hepatitis B patients.^[17–21]^ This protocol we drafted plans to assess the safety of Chinese compound prescriptions combined with nucleoside analogs in treating hepatic fibrosis in chronic hepatitis B patients and their adjuvant role in the combined therapy. The effective rate directly reflects the curative effect. Because liver biopsy is a kind of traumatic examination, most patients are reluctant to do so. Therefore, the combination of noninvasive biochemical parameters (hyaluronic acid, laminin, PC III, type IV collagen) and liver function indexes (alanine aminotransferase, aspartate aminotransferase, total bilirubin) is the main evaluation index for the diagnosis and prognosis of hepatic fibrosis. The level of HBV DNA indicates whether the patient has been infected by HBV and whether antiviral treatment is needed. Considering 2 different nucleoside analogs, ETV and TDF, we set up 2 prespecified subgroups. This protocol will conduct a meta-analysis of relevant randomized controlled trials and provide the latest evidence on the efficacy and safety of Chinese compound prescriptions combined with nucleoside analogs (ETV and TDF) in treating hepatic fibrosis in chronic hepatitis B patients, so as to better guide clinical practice.

## Author contributions

**Conceptualization:** Maoyuan Cheng, Baojia Wang.

**Data curation:** Maoyuan Cheng, Xianrong Feng.

**Formal analysis:** Baojia Wang.

**Methodology:** Li Ma.

**Project administration:** Baojia Wang.

**Software:** Maoyuan Cheng, Xianrong Feng, Long Wang.

**Supervision:** Yu Yang.

**Writing – original draft:** Maoyuan Cheng.

**Writing – review & editing:** Maoyuan Cheng, Baojia Wang.
